# Supplementary material for: Association between oral microbiome and seven types of cancers in East Asian population: a two-sample Mendelian randomization analysis
Source: Front Mol Biosci. 2023 Nov 21;10:1327893. doi: 10.3389/fmolb.2023.1327893 (PMC10702768; doi:10.3389/fmolb.2023.1327893)
Supplement: Supplementary file 1 [file DataSheet1.ZIP › Supplementary Materials/MR plots of saliva/saliva═╝/Breast cancer/pheno.3467_to_breast cancer_forest.pdf]

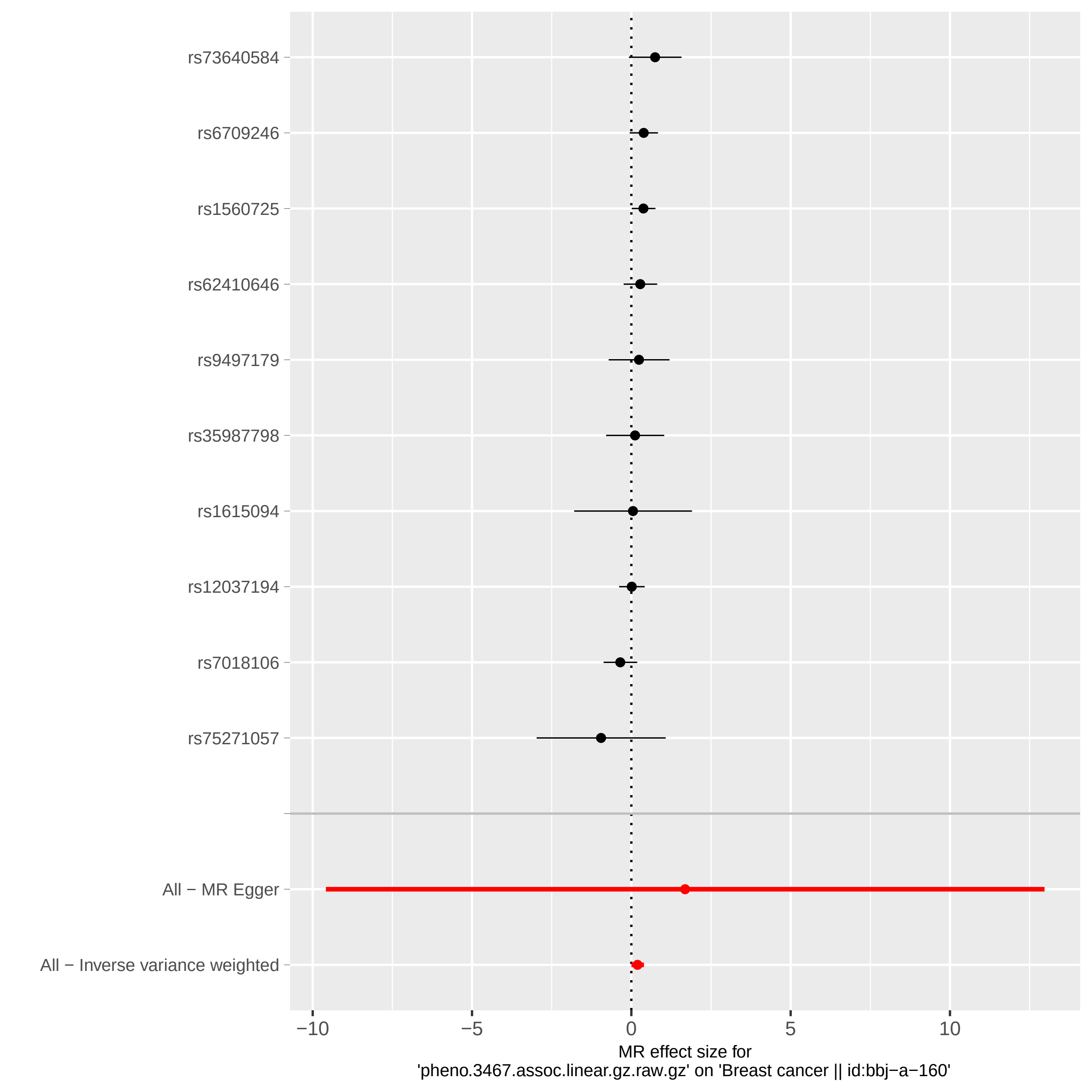
rs73640584
rs6709246
rs1560725
rs62410646
rs9497179
rs35987798
rs1615094
rs12037194
rs7018106
rs75271057
All − MR Egger
All − Inverse variance weighted
−10
−5
0
5
10
MR effect size for
'pheno.3467.assoc.linear.gz.raw.gz' on 'Breast cancer || id:bbj−a−160'
